# The Role of Nutrition in Neurological Disorders

**DOI:** 10.3390/nu15224713

**Published:** 2023-11-07

**Authors:** Gabriela Tsalamandris, Marios Hadjivassiliou, Panagiotis Zis

**Affiliations:** 1 Medical School, University of Cyprus, Nicosia 2029, Cyprus; gabriela.tsalamandris@gmail.com; 2 Academic Department of Neurosciences, Sheffield Teaching Hospitals NHS Foundation Trust, Sheffield S10 2JF, UK; m.hadjivassiliou@sheffield.ac.uk; 3 Medical School, University of Sheffield, Sheffield S10 2RX, UK

The interplay between nutrition and neurology has gained increasing recognition and various studies have emerged showing malnutrition and nutritional imbalances as a cause and result of certain neurological pathologies. Excessive or inadequate levels of certain nutrients as a result of malnutrition, illness, or the intake of certain drugs have been shown to cause or exacerbate certain neurological symptoms and disorders. Conversely, certain neurological conditions, like stroke, dementia, Parkinson’s disease (PD), and autism spectrum disorders, lead to a higher susceptibility to nutritional deficiencies, gastrointestinal disorders and feeding difficulties. This Special Issue of *Nutrients* explores the relationship between various nutrient imbalances and neurological conditions as well as the potential use of various nutraceuticals and dietary plans in the prevention and treatment of certain neurological disorders. Further, standardized scales and frequent screening in neurological patients with high susceptibility to nutritional deficiencies may assist physicians and caregivers in predicting, monitoring and preventing severe malnutrition and associated health problems, and their use in clinical practice is promising. 

It is widely recognized that both macronutrients and micronutrients play a vital role in the proper functioning of the nervous, cardiovascular, and immune systems, and changes in their levels, which may occur due to illness, malnutrition, or supplementation, can result in a broad range of clinical manifestations. A notable example of a disorder caused by malnutrition is Wernicke’s encephalopathy, a neurological disorder caused by thiamine (vitamin B) deficiency. Thiamine is crucial for several neuronal metabolic processes, and its depletion can result in neuronal cellular death and pathological lesions in various regions of the brain. The classical clinical symptoms of Wernicke’s encephalopathy include confusion, ocular irregularities, and ataxia, and are frequently accompanied by lesions in corresponding brain areas such as the thalamic nuclei, mammillary bodies, pontine tegmentum and cerebellar vermis. The heterogeneity of neuroimaging findings and clinical signs was emphasized in a case report and systematic review conducted by Cornea et al. which explored the prevalence of dysphagia in patients with Wernicke’s encephalopathy [1]. Out of the 13 patients with WE and dysphagia, 9 reported dysphagia at WE onset, while the classical triad of WE was seen in only three of them [1]. There were also differences in symptom onset and development, MRI findings and peripheral involvement amongst the reports [1]. It is believed that the distribution of lesions between alcoholic and non-alcoholic patients is different, with more frequent involvement of cranial nerve nuclei in non-alcoholic patients [1]. However, the reasons for these differences and the varying susceptibility of different brain regions to thiamine deficiency are not well known and more studies are needed to further our understanding of the effects of thiamine and other nutrient deficiencies on brain function.

Malnutrition and certain nutrient disturbances may also impact neurological function by affecting processes that are implicated in the development of cerebrovascular phenomena such as stroke [2,3,4]. Han et al. found that moderate to severely malnourished patients with acute ischemic stroke had a higher risk for stroke recurrence and major cardiovascular events, while the study by Chen et al. linked low nutritional status to a poorer prognosis in patients with severe stroke [2,3]. Both studies used serum albumin measurements to determine nutritional status and malnutrition risk, and its depletion is thought to be a sign of hyper-coagulation and high inflammation. Low serum albumin has long been associated with the pathogenesis of stroke and infection as it is thought to exert a neuroprotective function in stroke through antioxidant processes. Furthermore, fluctuations in selenium have also been indicated in stroke pathogenesis. The review paper by Liampas et al. demonstrated that both excess and decreased levels of selenium and selenoprotein expression can lead to immune and coagulation dysregulation [4]. Selenium has multiple pleiotropic homeostatic roles in human health and mediates its enzymatic functions through selenoproteins, which are known to moderate oxidative stress and coagulation processes such as the arachidonic cascade. Selenium’s protective role in cardiovascular disease has been proven in various studies showing an inverse relationship between low selenium levels and stroke, especially in elder, white, male populations. Furthermore, it has been shown that each 50 µg/L increase in blood selenium and in dietary selenium leads to a 38% and a 30% reduction in the prevalence of stroke, respectively [4]. Selenium supplementation in acute stroke patients has been shown to improve short-term acute stroke outcomes; however, there is limited evidence of its effects on long-term stroke outcomes, and more studies regarding its efficacy and safety must be conducted as selenium excess may cause dysregulation of glucose metabolism in addition to heightened immune activity [4].

The detrimental effects of disturbances in the gut–brain axis have also gained increasing recognition in the development and exacerbation of neurological and gastrointestinal disorders. The imbalance of intestinal microbiota can result from viral/bacterial infections, antibiotic use, and specific dietary regimens, and has been implicated in neuro-inflammatory processes that are linked to the pathogenesis of illnesses such as PD and Alzheimer’s Disease (AD) [5]. Conversely, neurological conditions such as PD, AD, multiple sclerosis, and chronic stress have been found to affect gut function and exacerbate inflammatory processes, including irritable bowel syndrome and leaky gut. [5] In the study conducted by Valenzuela-Zamora (2022), the relationship between food selectivity (and especially the elimination of nutrient-dense foods that modulate gut microbiota) in children with Autism Spectrum Disorder (ASD) and the development of gastrointestinal disorders (GID) was explored [6]. GIDs such as constipation, diarrhea, abdominal pain, and growth delays are reported in approximately 80% of children with ASD, and it is believed to be a result of intestinal dybiosis which leads to the increase in inflammatory bacteria such as Enterobacteriaceae, Salmonella Escherichia/Shigella, and Clostridium XIVa. The study hypothesized that these disturbances in the gut microbiota may contribute to issues related to behavior and cognitive performance while also exacerbating pre-existing GIDs. Other studies have also investigated the effects of food selectivity on the development of metabolic problems, such as obesity, in children with ASD. Raspini et al., 2022, used a semi-quantitative Food Frequency Questionnaire (FFQ) to investigate the dietary habits of children with ASD and found that they consumed more highly processed foods, simple sugars, and snacks when compared to their neurotypical peers [7]. The intake of fresh fruits and vegetables and complex carbohydrates was restricted and is thought to be a result of sensory aversion and neophobia which are common symptoms seen in ASD. While there was no statistically significant difference found between the body mass index (BMI) of children with ASD and that of their typically developing peers, there was significant evidence supporting the possibility of obesity and macronutrient deficiencies later in life as a result of these dietary habits [7]. These studies underscore the importance of frequent nutritional surveillance in high-risk neurological patients to ensure adequate nutritional intake and to prevent associated health problems.

In addition to poor dietary habits, nutritional disorders may also be caused by alterations in neurological processes involved in endocrine function and metabolism. For example, hereditary spastic paraplegia (HSP) is a neurodegenerative disorder characterized by spastic paraplegia; however, obesity and lymphedema are increasingly reported and are thought to be caused by hypothalamic adipocytokine resistance in patients with the SPG11 phenotype [8]. The study by Regensburger et al. explored the pathogenesis of obesity and lymphedema in patients with hereditary spastic paraplegia of the SPG11 allele and it was found that SPG11 HSP patients had increased tissue fat and fluid compartments and decreased hypothalamic volume with altered levels of adipocytokines. The role of the central nervous system in the etiopathogenesis of obesity in patients with SPG11 HSP was hypothesized due to the anatomical changes coupled with significantly elevated levels of leptin and resistin, despite the lipid profile of the patients being within physiological ranges. The hypothalamus and pituitary gland play a major role in adipose tissue homeostasis by secreting hormones and regulating satiety and energy expenditure, and neurometabolic alterations may have broad clinical signs, as seen in SPG11 HSP patients.

The effects of meal timing and nutrient intake distribution have gained importance in recent years, and the use of dietary plans, such as intermittent fasting for chronic pain treatment, has been tested [5]. The relationship between mealtimes and circadian rhythm in disease pathogenesis has also been shown in studies on chrononutrition, and it has been observed that eating at inappropriate times may lead to metabolic alterations and circadian rhythm desynchronisation, which are factors that play a role in the development of chronic disease and cognitive decline [5]. Conversely, changes in the distribution of calories throughout the day may be a marker of cognitive impairment [9]. This observation was derived from the Aiginition Longitudinal Biomarker Investigation of Neurodegeneration (Albion), in which thorough dietary and cognitive assessments were performed on non-demented participants aged >40 years with a positive family history of cognitive decline or a concern for cognitive status. The results of the study show that individuals who consume high-energy meals at the beginning of the day have a better cognitive status than those whose meal patterns are characterized by low-energy meals oriented towards the end of the day. Brikou et al. hypothesized that this may be because individuals with poor cognitive function opt to eat earlier in the day when they have better cognitive performance. This preponderance of calories consumed earlier in the day was also observed in a similar study involving seniors with Alzheimer’s disease and behavioral difficulties, highlighting the role of circadian desynchronization and mental disorganization in the diet.

Drugs may cause fluctuations in nutrients levels which may lead to a broad range of neurological symptoms. An example of this can be seen in the study by Matsuyama et al. (2021) which showed a negative correlation between levadopa, a zinc-chelating drug, and serum zinc levels [10]. Zinc, a micronutrient involved in the functions of the integumentary, immune, and nervous system, was observed to be lower in Parkinson’s patients taking levodopa, resulting in taste disorders, stomatitis, dermatitis and psychiatric symptoms. Zinc supplementation led to the improvement of taste disorders and dermatitis in 15 of the 27 patients with low serum zinc levels (<80 µg/dL); however, no significant improvement was observed in the MDS-UPDRS, PDQ39 score, and H-Y stage. It was also hypothesized that zinc deficiency may lead to the exacerbation of psychiatric symptoms such as depression, anxiety, and sleep disorders, presumably due to zinc’s anti-inflammatory and antioxidant effects and its involvement in the regulation of brain receptors such as serotonin and glutamate receptors [10].

Supplementation and drug intake can also result in excessive nutrient levels, which may cause or exacerbate an underlying neurological disorder. Excessive levels of vitamin B6, usually a result of dietary supplementation, have been shown to cause sensory peripheral neuropathy of the axonal type [11]. Daily intake of B6 exceeding 100 mg/day, the upper limit set by the FDA, either in the form of a multivitamin or by supplementation with pyridoxine (one of its three naturally occurring forms) was shown to cause polyneuropathy in various patient groups included in a systematic review conducted by Muhamad et al., 2023 [11]. Although resynchronization or improvement of symptoms following discontinuation of B6 supplementation has been reported, there are few reports of symptom resolution and little evidence regarding follow-up electrophysiological findings. Conversely, low levels of vitamin B6 can be seen in patients with peripheral neuropathy of various etiologies; however, the direct causal relationship between B6 deficiency and PN is not fully understood, as these patients often have a poor overall nutritional status (and thus may have deficiencies in other nutrients, such as B12) and/or receive treatments that can lead to neuropathy for conditions such as diabetes or chronic renal failure. There are limitations in our understanding of the exact effects and mechanisms of drug-related nutrient imbalances on neurological conditions and these studies emphasize the need for further research as well as the importance of frequent monitoring and high clinical suspicion in susceptible patient groups. 

Although the use of certain foods and diets is yet to be widely implemented, various nutraceuticals have been shown to have great potential in the prevention and treatment of many neurological conditions [5,12]. Numerous studies have proven that nutrient-dense diets, such as the Mediterranean diet or diets high in omega-3 fatty acids, have neuroprotective and cardiometabolic benefits, while diets high in processed foods and saturated and trans fats are thought to play a role in low-grade systemic inflammation, which in turn contributes to the neuro-inflammatory mechanisms involved in the pathogenesis of degenerative diseases and depression [5]. There is increasing evidence of the potential use of vitamin D supplementation in patients with Alzheimer’s disease, Parkinson’s disease, and other neurological diseases as low levels of vitamin D have been linked to a higher risk of cognitive decline. Other examples of potential neuronutritional interventions include nicotinamide riboside supplementation for the treatment of PD clinical signs; pro/prebiotics for the prevention of Alzheimer’s disease; and magnesium, coenzyme Q10, feverfew, riboflavin, and phycocyanins for migraine treatment [5]. The use of a ketogenic diet for drug-resistant epilepsy and glucose transporter type 1 (GLUT1) deficiency syndrome has also become more widespread in recent years owing to metabolic alterations and neuroprotective mechanisms linked with ketogenesis. Ketone bodies, derived from fatty acid oxidation, a process that occurs during fasting states or as a result of high-fat diets, have broad antioxidative and neuroprotective properties and are thought to prevent neuronal cell damage following epileptic seizure [12]. Additionally, the KD is thought to create an anti-seizure state by increasing inhibitory effects mediated through adenosine A1 receptors as well as by enhancing chemical messengers in the brain such as GABA [12]. The therapeutic role of vitamin B6 supplementation in patients with peripheral neuropathy has been suggested in many studies; however, the exact efficacy is yet to be understood, because B6 was administered as part of a combination treatment containing other vitamins and not as a monotherapy [12]. Further research is needed to understand the exact relationship between peripheral neuropathy and vitamin B6 excess and deficiency, and its potential use as a supplement for the treatment of neuropathic symptoms. More extensive studies on the efficacy and safety of these interventions are needed, but there is substantial evidence supporting the value of neuronutrition for treatment and disease prevention.

Malnutrition is a common risk in neurological patients due to feeding difficulties and/or therapeutic interventions coupled with their inability to communicate or take care of their dietary requirements, and thus frequent monitoring and the use of predictive tools should be implemented to assist caregivers and health professionals. The observational prospective study by Gorska et al. demonstrated the high frequency of undernourishment and hypoglycemia in children with severe central nervous system impairment and highlighted the need for frequent monitoring of nutritional status [13]. CNS impairment is frequently associated with gastrointestinal disorders such as gastro-esophageal reflux, dumping syndrome and constipation, and it is believed that these comorbidities increase the risk of malabsorption and hypoglycemia despite enteral feeding with standardized manufactured formulas [13]. Hunger or signs of hypoglycemia may be difficult to identify in patients with severe CNS impairment due to communication difficulties and cognitive impairment, and thus the use of tools such as continuous glucose monitoring (CGM) may be useful in ensuring adequate nutritional support to these patients. Prognostic scales and screening tools like CONUT and the GNRI may be helpful to physicians in identifying high-risk patients following stroke. The geriatric nutritional risk index (GNRI) is an index integrating body mass index (BMI) and serum albumin, and its prognostic potential was shown in the study by Chen et al. where low GNRI levels were associated with higher mortality rates in severe stroke patients [3]. The GNRI is more easily measurable compared to other nutritional scales like NUTRIC and NRS2002, which may be difficult to perform on patients with cognitive impairment or sensory deficits, and GNRI implementation in clinical practice may assist physicians in their treatment approach [3]. Additionally, the risk of stroke recurrence can be determined using certain nutritional indices like the controlling nutritional status (CONUT) score and the prognostic nutritional index (PNI) [2]. The CONUT and PNI scores are nutritional indices that can be calculated from routine blood-based tests (including total peripheral lymphocytes count, serum albumin, and total cholesterol), and the potential for their use as predictors of recurrent ischemic stroke (RIS) in patients with acute ischemic stroke was demonstrated by Han et al. [2]. In addition to frequent clinical monitoring, the use of standardized dietary guidelines and nutritional educational programs may help mitigate nutritional deficiencies in neurological patients with high susceptibility. The scoping review by Russell et al. indicated, the lack of nutrition education programs for adults with neurological diseases and that the few existing programs do not meet the best practice principles for nutrition education regarding delivery, educator characteristics, and evaluation [14]. It was demonstrated that many of the disease-specific nutritional programs (targeted to dementia, PD and stroke patients) did not incorporate behavior change theory, and evidence of co-design with patients was lacking [14]. As malnutrition poses a great risk in neurological patients, the need for the development of more effective and holistic disease-specific educational programs for neurological patients and their caretakers is becoming increasingly evident, and much progress is needed in this aspect of neuronutrition.

With the furthering of our understanding of the overlap between nutrition and neurology, there are numerous neuronutritional interventions that show great promise in combating neurological conditions. The need for surveillance tools and dietary guidelines is evident due to the increased risk of malnutrition in neurological patients.
